# RETRACTION: Sunflower Pollen and Bumble Bee Health: Mechanisms, Modifiers and Trade‐Offs

**DOI:** 10.1002/ece3.74178

**Published:** 2026-08-17

## Abstract

**RETRACTION**: OdemerR. , “Sunflower Pollen and Bumble Bee Health: Mechanisms, Modifiers and Trade‐Offs,” Ecology and Evolution 16, no. 2 (2026): e73107, 10.1002/ece3.73107.41710518 PMC12910244

The above article, published online on 17 February 2026 in Wiley Online Library (wileyonlinelibrary.com), has been retracted by agreement between the journal Editor‐in‐Chief, Arley Muth; and John Wiley & Sons, Ltd. The retraction has been agreed following concerns raised by a third party. An investigation identified multiple instances where statements in the review article were linked to citations that were not sufficiently specific to support the claims being made. In some cases, the cited references did not address the topic under discussion, while in others the findings reported in the cited literature differed from those described in the article. The author cooperated with the investigation, acknowledged citation and wording errors, and proposed corrections. However, the extent of the amendments required, including changes affecting the article's conclusions, was considered to require reframing of the article beyond what could reasonably be addressed through a correction. The editors therefore no longer have confidence in the reliability of the article. The author disagrees with the decision to retract the article and with the wording of the retraction notice.

**RETRACTION**: R. Odemer , “Sunflower Pollen and Bumble Bee Health: Mechanisms, Modifiers and Trade‐Offs,” Ecology and Evolution 16, no. 2 (2026): e73107, 10.1002/ece3.73107.41710518 PMC12910244

The above article, published online on 17 February 2026 in Wiley Online Library (wileyonlinelibrary.com), has been retracted by agreement between the journal Editor‐in‐Chief, Arley Muth; and John Wiley & Sons, Ltd. The retraction has been agreed following concerns raised by a third party. An investigation identified multiple instances where statements in the review article were linked to citations that were not sufficiently specific to support the claims being made. In some cases, the cited references did not address the topic under discussion, while in others the findings reported in the cited literature differed from those described in the article. The author cooperated with the investigation, acknowledged citation and wording errors, and proposed corrections. However, the extent of the amendments required, including changes affecting the article's conclusions, was considered to require reframing of the article beyond what could reasonably be addressed through a correction. The editors therefore no longer have confidence in the reliability of the article. The author disagrees with the decision to retract the article and with the wording of the retraction notice.

